# Role of Probiotics and Prebiotics in Gut Symbiosis

**DOI:** 10.3390/nu16020238

**Published:** 2024-01-12

**Authors:** Steven T. Leach

**Affiliations:** Department of Paediatrics, School of Clinical Medicine, University of New South Wales, Sydney 2052, Australia; s.leach@unsw.edu.au

The objective of this Special Issue entitled ‘Role of Probiotics and Prebiotics in Gut Symbiosis’ is to publish reviews, clinical trials and experimental studies that focus on probiotics and prebiotics that have a role in influencing disease and promoting gastrointestinal and overall health. It is often stated that gastrointestinal health is essential to the overall health of the individual. In addition, with the increasing use of the concept of dysbiosis, it has been found that dysbiosis of the microorganisms in our gut (our microbiome) is bad for overall health. But what is dysbiosis? To completely appreciate the concept of dysbiosis, we should also be familiar with the opposing concept of symbiosis. Symbiosis can be defined as a relationship between two different living organisms that cohabitate and provide mutual benefits to each other. With this definition in mind, the gastrointestinal tract of a healthy human can be considered to be in a symbiotic relationship with the gastrointestinal microbiome. Investigating this further, one can ask what mutual benefits the host and the microbes receive. The primary benefit that the intestinal microbiome receives is that the host provides it with a warm stable environment, and with a continual supply of nutrients. This is very beneficial for the survival of these microbes indeed! But what does the host receive? The microbiome can assist in the breakdown of nutrients which the host does not have the biochemical capacity to utilise. The result of this microbial breakdown and biochemical processing is that additional nutrients become available to the host [1]. Although this is somewhat beneficial, on face value, the contribution of the intestinal microbiome does not appear essential to the survival of the host. Furthermore, this benefit may be considered subjectively less beneficial than the benefit that the microbiome receives, and one could even consider that the host may be better off with no microbiome at all. However, there is another benefit that the host receives that is not immediately apparent, and only becomes apparent when lost. This is where the concept of dysbiosis is introduced, and where ‘the loss of symbiosis’ indicates that the gastrointestinal microbiome has an overall benefit that impacts the survival of the host.

Sun Tzu, the 6th century Chinese military general and strategist, is quoted as saying “Keep your friends close and enemies closer”. Although this may be a wise teaching for dealing with military foes, for human–microbial interactions, an alternative strategy of keeping your “friends close and your enemies far away” may be preferable. That is, in a world full of microbial nasties, having a gut full of ‘friendly’ bacteria can help protect you from your bacterial enemies. So what happens when symbiosis is lost? When relationships break down, often the opposing parties will attack each other, and in the case of host–microbial interactions, this manifests as disease. Dysbiosis has been implicated in several diseases, most notably inflammatory disease [2] (with the host on the attack), but also psychological manifestations through the gut–brain axis [3]. The concept of the gut–brain axis, which describes a bi-directional communication between the intestine and the brain [4], affects emotions, mood and cognitive functions and hence can impact not only health but also host survivability [5]. Thus, a good symbiotic ‘relationship’ with one’s gut bacteria can be considered an essential benefit for the host as it potentially impacts survivability.

Investigating dysbiosis further, the concept of dysbiosis as the loss of symbiosis remains valid. That is, if your microbiome is not helping you, it is hurting you. In health, the current overarching belief is that if dysbiosis is present, health can be improved through correcting the dysbiosis via promoting a state of symbiosis [6]. This is where probiotics and prebiotics are viewed as prime candidates to assist in promoting a state of gut symbiosis and promoting gut health.

A further example of where the use of probiotics to prevent dysbiosis and disease to assists in the survivability of the host is in the use of probiotics in newborn premature infants [7]. Prematurity leads to a significant risk of developing necrotizing enterocolitis (NEC) early in life [8], and once NEC has developed, the infant is at significant risk of morbidity and mortality [9]. NEC is a syndrome with a proposed aetiology of intestinal microbiota stressing an immature gastrointestinal tract with a precarious vascular supply, which cascades to acute intestinal ischemic necrosis [10]. The contribution of gastrointestinal bacteria and the protective effects of probiotics are demonstrated by a meta-analysis of NEC/probiotic clinical trials in neonates where the authors conclude that probiotics effectively reduce the occurrence and mortality of NEC [11]. Although this meta-analysis is promising, a further meta-analysis arrives at more guarded conclusions when the trial cohorts focused on very and extremely low-birth-weight infants, albeit with a mix of probiotic strains across the trials [12]. Nevertheless, the evidence indicates that in neonates, receiving beneficial bacteria can impact survivability. However, the precise effects of differing probiotic species in differing infant cohorts requires more thorough investigation to be optimally integrated into clinical practice.

In summary, there is an accumulating body of evidence showing that probiotic bacteria, and the prebiotic components that support the growth and function of probiotic bacteria, can offer substantial and measurable benefits to human health. However, for probiotics and prebiotics to be utilised with optimal efficacy there remains many questions: what are the best pre/probiotics? Do pre/probiotics work the same for everyone, and for which conditions? Pre/probiotics are generally thought to be safe in the short term, but what are the long-term risks? These are but a few of the important questions we must both ask and answer.

## References

[1] Sikorska-Zimny K., Beneduce L. (2021). The Metabolism of Glucosinolates by Gut Microbiota. Nutrients.

[2] Dahal R.H., Kim S., Kim Y.K., Kim E.S., Kim J. (2023). Insight into gut dysbiosis of patients with inflammatory bowel disease and ischemic colitis. Front. Microbiol..

[3] Mohan A., Godugu S., Joshi S.S., Shah K.B., Vanka S.C., Shakil H., Dhanush P., Veliginti S., Sure P.S., Goranti J. (2023). Gut-brain axis: Altered microbiome and depression—Review. Ann. Med. Surg..

[4] Chaudhry T.S., Senapati S.G., Gadam S., Mannam H., Voruganti H.V., Abbasi Z., Abhinav T., Challa A.B., Pallipamu N., Bheemisetty N. (2023). The Impact of Microbiota on the Gut-Brain Axis: Examining the Complex Interplay and Implications. J. Clin. Med..

[5] Garvey M. (2023). The Association between Dysbiosis and Neurological Conditions Often Manifesting with Chronic Pain. Biomedicines.

[6] Dixit K., Chaudhari D., Dhotre D., Shouche Y., Saroj S. (2021). Restoration of dysbiotic human gut microbiome for homeostasis. Life Sci..

[7] Mercer E.M., Arrieta M.C. (2023). Probiotics to improve the gut microbiome in premature infants: Are we there yet?. Gut Microbes.

[8] Gephart S.M., McGrath J.M., Effken J.A., Halpern M.D. (2012). Necrotizing enterocolitis risk: State of the science. Adv. Neonatal Care.

[9] Duess J.W., Sampah M.E., Lopez C.M., Tsuboi K., Scheese D.J., Sodhi C.P., Hackam D.J. (2023). Necrotizing enterocolitis, gut microbes, and sepsis. Gut Microbes.

[10] Tanner S.M., Berryhill T.F., Ellenburg J.L., Jilling T., Cleveland D.S., Lorenz R.G., Martin C.A. (2015). Pathogenesis of necrotizing enterocolitis: Modeling the innate immune response. Am. J. Pathol..

[11] Wang H., Meng X., Xing S., Guo B., Chen Y., Pan Y.Q. (2023). Probiotics to prevent necrotizing enterocolitis and reduce mortality in neonates: A meta-analysis. Medicine.

[12] Sharif S., Meader N., Oddie S.J., Rojas-Reyes M.X., McGuire W. (2020). Probiotics to prevent necrotising enterocolitis in very preterm or very low birth weight infants. Cochrane Database Syst. Rev..

